# Scientific Evidence and Potential Barriers in the Management of Brazilian Protected Areas

**DOI:** 10.1371/journal.pone.0169917

**Published:** 2017-01-09

**Authors:** Eduardo L. H. Giehl, Marcela Moretti, Jessica C. Walsh, Marco A. Batalha, Carly N. Cook

**Affiliations:** 1 Department of Ecology and Zoology, Federal University of Santa Catarina, Florianópolis, Santa Catarina, Brazil; 2 Department of Botany, Federal University of São Carlos, São Carlos, São Paulo, Brazil; 3 Environmental Management and Analysis, Federal University of São Carlos, São Carlos, São Paulo, Brazil; 4 Department of Zoology, University of Cambridge, Cambridge, United Kingdom; 5 School of Biological Sciences, Monash University, Clayton, Australia; University of Brighton, UNITED KINGDOM

## Abstract

Protected areas are a crucial tool for halting the loss of biodiversity. Yet, the management of protected areas is under resourced, impacting the ability to achieve effective conservation actions. Effective management depends on the application of the best available knowledge, which can include both scientific evidence and the local knowledge of onsite managers. Despite the clear value of evidence-based conservation, there is still little known about how much scientific evidence is used to guide the management of protected areas. This knowledge gap is especially evident in developing countries, where resource limitations and language barriers may create additional challenges for the use of scientific evidence in management. To assess the extent to which scientific evidence is used to inform management decisions in a developing country, we surveyed Brazilian protected area managers about the information they use to support their management decisions. We targeted on-ground managers who are responsible for management decisions made at the local protected area level. We asked managers about the sources of evidence they use, how frequently they assess the different sources of evidence and the scientific content of the different sources of evidence. We also considered a range of factors that might explain the use of scientific evidence to guide the management of protected areas, such as the language spoken by managers, the accessibility of evidence sources and the characteristics of the managers and the protected areas they manage. The managers who responded to our questionnaire reported that they most frequently made decisions based on their personal experience, with scientific evidence being used relatively infrequently. While managers in our study tended to value scientific evidence less highly than other sources, most managers still considered science important for management decisions. Managers reported that the accessibility of scientific evidence is low relative to other types of evidence, with key barriers being the low levels of open access research and insufficient technical training to enable managers to interpret research findings. Based on our results, we suggest that managers in developing countries face all the same challenges as those in developed countries, along with additional language barriers that can prevent greater use of scientific evidence to support effective management of protected areas in Brazil.

## Introduction

Protected areas are important to conserve biodiversity and maintain a wide range of ecological processes and ecosystem services [1–3]. Nevertheless, protected areas face substantial threats [2], challenging their effectiveness as a tool for conservation [4]. Improving this scenario is a substantial challenge when only 20–40% of the world’s protected areas are considered to be managed effectively (i.e. achieving their stated objectives through effective management) [5].

Effective management of protected areas depends on the application of successful management actions. Selecting the most appropriate management actions requires a sound evidence base, derived from scientific evidence and the experience that managers acquire during their daily work [6]. Although managers can benefit from access to a range of different types of evidence when making management decisions [7], the few studies that have examined the use of evidence by managers have reported that most managers rely solely on their own experience [8–10]. The limited assessment of the use of evidence by managers has overwhelmingly been focused on English speaking countries, such as Australia [8], United Kingdom [6], United States [10], and South Africa [11]. We know of only one study that has considered the use of evidence by protected area managers in other parts of the world [12], leaving a substantial gap in our understanding of extent of evidence-based practice in the non-English speaking world.

There are many reasons why managers tend not to use scientific evidence [13], including the difficulty accessing and interpreting evidence [6,9], the urgent nature of many decisions being incompatible with the time required to search for scientific evidence [8,14], and a lack of relevant science [15,16]. However, given the bias in our understanding of evidence-based conservation towards the English speaking world, it is highly likely that managers from non-English speaking countries face additional challenges when seeking access to scientific evidence due to language difficulties (e.g. [17,18]). This bias also means that our understanding of evidence-based management is weakest in parts of the world where many of the biodiversity hotspots are concentrated. Therefore, there is an urgent need to understand the use of scientific information in these regions of the world.

Understanding the types of evidence managers use to support their decisions, and why, can help to reveal the barriers they face in utilizing the best available evidence. It can also provide insights into how to design strategies to help managers achieve greater access to the evidence most valuable for their decisions. For example, the strategies to support evidence-based conservation would be different if managers have limited access to scientific evidence versus if they have insufficient training to understand the relevant science. Thus, our main contribution is both to provide information on the type of evidence managers from a non-English speaking country use to make decsions and to understand the barriers that may prevent managers from using science more frequently.

To understand the use of scientific evidence by protected area managers in a non-English speaking country, we spoke with managers of protected areas in Brazil. Brazil has one of the largest protected area networks in the world [19] encompassing an important global biodiversity hotspot. Brazil hosts over 8% of the world’s vascular plants, half of which are endemic [20]. We were interested not only in the types of evidence being used by managers to support their decisions, but also in the scientific rigour associated with the different sources of information and how frequently managers were using these different sources of evidence. We combined the scientific rigour of the information and frequency with which managers used different types of evidence into an index representing the degree of evidence-based decision making (EBDM score). This measure may not capture the overall complexity involved in the way managers may use science under all circumstances. However, both pieces of information are important to capture because even if managers value science and use it to support their management decisions, when they access it rarely, science is unlikely to make a substantial contribution to evidence-based decision making.

As one of the first studies to investigate the extent of evidence-based decision making in the non-English-speaking world, we sought to understand: 1) where managers look for information, 2) what drives their choice in looking at these sources, 3) what types of evidence they consider valuable for decisions, 4) what they can access, and 5) which factors most influence the use of science by managers.

## Materials and Methods

### Study system

Brazil has 1,940 protected areas, varying in size, administrative divisions, and protection type (S1 Table and S1 Fig [21]). The overall terrestrial area protected exceeds 220 million hectares [22], covering over 18% of the land area, much of which is accounted for by large protected areas in northern regions (S1 Fig). There are 954 federal, 781 state and 205 municipally managed protected areas and governance (e.g., resourcing) can vary significantly between the different administrative divisions, but detailed information is lacking.

### Data collection

#### Survey

We obtained contact details for 1,040 managers responsible for the day-to-day management of Brazilian protected areas from the Brazilian Ministry of the Environment. These individuals represent the managers responsible for at least one protected area within one of the three different administrative divisions (federal, state, and municipality). The protected areas included all protection categories (strict protection, sustainable use, and further subdivisions; S1 Table). We contacted each of these managers by e-mail, explaining the reason for the inquiry and the aims of our study, and provided them with a link to an online survey (S1 Text). We collected data anonymously and all respondents were asked to read a statement where they were informed about the procedures and then decide whether to answer the questionnaire. Acceptance to the conditions and proceeding to answering the questionnaires was considered as given consent. We sent three follow up reminders to increase the number of respondents [23] over a four month period. This study was registered and approved by the National Bioethics Commission of Brazil (CONEP–“Comissão Nacional de Ética em Pesquisa”) with the ID: CAAE 12964013.0.0000.5504.

The survey was divided into two sections. In the first section, we collected general information about the protected area (regional location, size, and protection category), and about the manager, including his/her level of experience (number of years as a protected area manager), preliminary technical training (whether the manager received any kind of training directed to protected area management), education level (formal qualifications), English fluency, and their role in the protected area (advising, decision-making, or implementing actions). We then provided respondents with a list of 21 different sources of evidence (Table 1) and ask them to rate: i) how important they believed each source to be for making good decisions (on a 3-point scale; Table 2), ii) how frequently they used each of the information sources (on a 5-point scale; Table 2) and iii) how accessible they believed each information source to be (on a 4-point scale; Table 2). The order of the list of sources was randomized for each question, and between respondents, to avoid potential biases in the answering process. The range of information sources presented to respondents was derived from a combination of lists used in previous studies (e.g. [6–8,10,11,18]). In the third section, we asked managers to indicate the factors that they considered while choosing information to support management decisions. The list of potentially important or limiting factors was compiled from the literature and covered issues related to either the ability to access and interpret information, or the credibility of any of the sources used in their management decisions (S2 Table).

**Table 1:** Type of information sources included in the questionnaires answered by Brazilian protected area managers, classified according to the level of scientific rigour (scientific foundation score) and categorised as scientific evidence, intermediate evidence and experience-based evidence.

Evidence category	Source	Median rank	Scientific foundation score*
Scientific	Scientific research papers	1	1
	Science magazines	2	0.95
	Books or book chapters	4	0.86
	Policy-briefing documents and technical information leaflets	5	0.81
	Unpublished theses	7	0.71
Intermediate	Advice from experts or scientists outside organization	8	0.67
	Internal databases, archives and records	8	0.67
	Management plans, working manuals and guidelines	8	0.67
	Published reports	9	0.62
	Conference proceedings or presentations	10	0.57
	Seminars and workshops	11	0.52
	Specific environmental websites, databases or web tools	12	0.48
	Training courses	12	0.48
	E-bulletins or newsletters	14	0.38
Experience	Own field based knowledge, observations and experience	14	0.38
	Site visits or short term staff exchanges	15	0.33
	Advice from colleagues or experts within organization	16	0.29
	Local knowledge and observations from community members	16	0.29
	Informal discussion with colleagues	17	0.24
	Public media, e.g. newspapers, television, films, radio	20	0.1
	YouTube videos or podcasts	21	0.05

* The scientific foundation scale (SFS) was determined by the median of ranks assigned by an expert panel, where experts were asked to order sources based on the level of scientific rigour each contained (see Expert elicitation section for details). This measure was subsequently used as a weighting to determine the evidence-based decision making scores.

**Table 2:** The coding system used to score managers’ responses to questions about the frequency of use, accessibility and importance of each source of evidence.

Frequency of use	Code	Accessibility	Code	Importance	Code
At least once a week	1000	Easily accessible	3	Very important	3
At least once a month	100	Accessible with slight difficulty	2	Important	2
At least once a year	10	Accessible with moderate or great difficulty	1	Little importance	1
Less than once a year	1	Not accessible	0		
Never	0	Don’t know	NA		

#### Expert elicitation

To classify the different types of evidence into categories that represent the scientific rigor of the sources, we assembled an expert panel of 19 Brazilian researchers and protected area managers. The researchers were ecologists and conservation scientists holding positions at Brazilian universities and randomly selected from the Brazilian national résumé database (“Currículo Lattes”; http://lattes.cnpq.br/) and the managers were a sub-sample of the respondents of our main survey. The expert elicitation process was carried out with an online survey. The members of the expert panel were first asked to allocate each of the 21 sources of evidence (Table 1) to one of three information types (adapted from [8]): i) scientific evidence, representing sources with strong scientific foundations; ii) intermediate evidence, a mixture of scientific evidence, raw data and reports, and experience; and iii) experience-based evidence, representing personal observations and practical experience. The order of the list of sources was randomized to prevent biases in the answering process.

Next, the experts were asked to rank the information sources by their scientific rigor. The median rank attributed to each source was then calculated and scaled to a value between 0–1 by applying the following transformation: *Score*_*n*_ = ((*n* + 1) − *r*_*n*_)/*n*, where *n* is the number of sources and *r* is the rank of the source *n*. We called this the “scientific foundation scale” where values close to one indicate a strong scientific foundation (e.g., peer-reviewed) and values close to zero indicate a poor scientific foundation (e.g., information that cannot be traced to the original source). The median ranks, the final ordering and scores are provided in Table 1.

### Data analyses

#### Where do managers go to find information?

To assess where managers go to find information about management decisions, we grouped sources into the categories of evidence assigned by the expert panel (scientific evidence, intermediate evidence, or experience-based evidence). Then, to determine if there are categories of evidence managers consult most frequently, we used Analysis of variance (ANOVA) to evaluate which types of evidence were reportedly used more frequently. Managers’ responses in relation to the frequency of use were assigned a numeric code (Table 2); we used a log scale to reflect the fact that the categories we used were not linear, with weekly use (frequency score = 1,000) being much more frequent than monthly use (frequency score = 100), which is much more often than yearly use (frequency score = 10) and so on. The coded frequency of use scale (Table 2) was used as a continuous response variable with evidence category as a categorical predictor variable.

#### What influences the likelihood that managers use scientific evidence?

To assess the degree to which managers use scientific evidence, were used (ANOVA) to determine whether managers reported difference in the accessibility and importance of different categories of evidence. The coded accessibility scale and importance scores (Table 2) was used as continuous response variables with evidence category as a categorical predictor variable. Separate analyses were run for the accessibility of evidence and the level of importance of evidence.

To understand what influences whether or not managers use scientific evidence, we calculated a metric to indicate the degree of evidence based decision making by each manager (EDBM score). To do this, we combined data on how frequently managers use each of the sources of evidence (Table 2) with the level of scientific rigour (scientific foundation score) of each of the sources (Table 1). To combine the scientific rigour with the frequency with which sources were used, we multiplied the scientific foundations scale by the frequency of use scale, creating an index of evidence-based decision making (EBDM score). The EBDM score was calculated for each of the managers in our sample, for each information source (Table 1) in each of the evidence categories (scientific evidence, intermediate evidence, and experience-based evidence) as:

$${EBDM}_{i}=\frac{1}{m}\sum_{c=1}^{m}\frac{1}{n}\sum_{s=1}^{n}{uf}_{is}\times {sf}_{s}$$

where the EBDM score of manager *i* is the mean of all *m* evidence categories and where the values for each evidence category *c* are the mean product of the use frequency *uf* by the scientific foundation *sf* of information source *s* across all *n* information sources of that evidence category. These EBMD scores provided us with a measure of how often managers are using the most rigorous evidence in each evidence category when making their decision, which we could then use to evaluate the factors that might explain the uptake of scientific evidence by managers.

To understand which variables contribute to difference in the use of evidence by managers we used a general linear model, testing the mean EBDM scores (response variable) against a range of different explanatory variables. The EBDM scores were subjected to a ¼-power transformation to meet the assumptions of normality and equal variance of residuals required by linear regression. The explanatory variables we tested included characteristics of the manager: their level of experience (years), preliminary technical training, education level, and English fluency. We also assessed the influence of protected area characteristics, such as the geographic location, size, and protection category of the different protected areas. Finally, we tested the influence of the accessibility and perceived level of importance of the information sources as indicated by managers (Table 2). Next, we applied a step-by-step variable selection procedure, starting with the full model and then excluding variables based on changes to Akaike information criterion (AIC). Overall, we selected the model with the lowest AIC. All analyses were run in R [24].

## Results

We received 282 responses from managers with a response rate of 27%, of which 233 answered all questions (22% response rate). This is a slightly higher response rate than similar studies on protected area managers [7] and is likely to be an underestimate because it was not possible to estimate how many of the questionnaires did not reach managers who were on leave at the time of the survey. Approximately 87% of managers indicated that their roles included implementing conservation actions; 63% said they were decision-makers in one or multiple protected areas; and 23% indicated they had an advisory role, with many managers indicating multiple responsibilities (Table 3). On average, managers had 6.7 years of experience (median = 5 years; range 4 months to 30 years). Most managers had either finished tertiary education at a university (39%), had a Master’s degree (30%), or had undertaken some form of specialization after tertiary education (23%; Table 3). Most managers described their English language skills as elementary (43.8%) or intermediate (31.7%), with more than 50% of managers in the lowest two fluency categories (Table 3). Of the responding managers, 57% worked in protected areas with strict protection and 43% in protected areas allowing sustainable use of natural resources (S1 Table). The median size of protected areas was 9,950 ha, ranging from 2.7 ha up to six million ha.

**Table 3:** Description of the sample of managers who responded to the questionnaire regarding their standard of English fluency, role in protected area, and education level.

English skills		Role in protected area^a^		Education level	
Advanced	45 (17%)	Advisor	62 (23.2%)	Doctorate	15 (5.6%)
Intermediate	84 (31.7%)	Decision-maker	168 (62.9%)	Masters	81 (30.5%)
Elementary	116 (43.8%)	On-ground management	233 (87.3%)	Specialization^b^	60 (22.6%)
Beginner	20 (7.5%)			Tertiary education^c^	104 (39.1%)
				Technical school	4 (1.5%)
				Secondary school	2 (0.8%)

^a^ More than one role could be played by the same manager in a protected area and therefore the sum of percentages for Role in protected area exceeds 100%.

^b^ Obtained after tertiary education, but not equivalent to a Master’s degree because of shorter duration or less rigorous scientific demands. These courses are normally taken in between tertiary education and a Master’s degree.

^c^ Equivalent to a Bachelor’s degree or a diploma in any field, but obtained in a university.

(n = 267; the number of managers who answered the demographic questions in the questionnaire)

### Where do managers go to find information?

**Fig 1:**
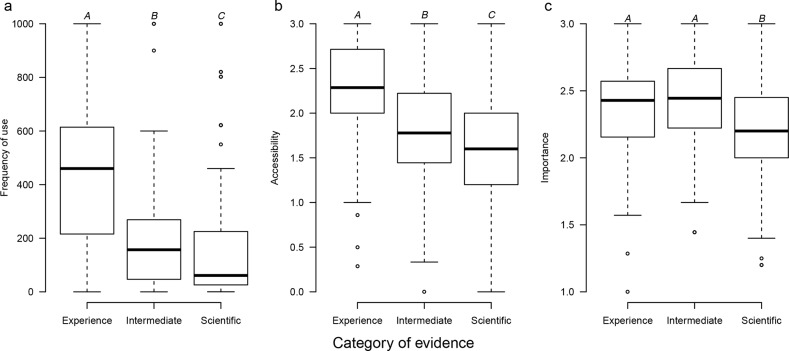
Frequency of use, accessibility, and importance of evidence categories reported by Brazilian managers of protected areas. (a) Frequency of use by evidence category (scientific evidence, intermediate and experience). (b) Accessibility by evidence category. (c) Importance by evidence category. All three variables differed among evidence categories. Letters above boxplots indicate which pairwise comparisons were significantly different after Tukey tests.

When considering information sources managers were significantly more likely to use experience-based evidence with lower levels of use of intermediate and scientific evidence (F_2, 776_ = 162.2, P < 0.001; Fig 1A). Experience-based evidence was 3.1 and 2.3 times more frequently used than scientific and intermediate evidence, whereas intermediate evidence was used 1.4 times more frequently than scientific evidence (experience: $\bar{x}$ = 445.2; intermediate evidence: $\bar{x}$ = 194.6; scientific evidence: $\bar{x}$ = 143.4). Generally, the sources of experience-based evidence were the field based knowledge and observations of managers (~71% of managers; S2B Fig), or discussions with their colleagues (~63%; S2B Fig). The most commonly used intermediate sources were protected area databases (43%; S2B Fig), or specific environmental websites (41%; S2B Fig). Finally, the most commonly used sources (i.e., on a weekly basis) with the highest scientific content were books or book chapters (13%, S2B Fig), and policy-briefing documents and technical leaflets (24%; S2B Fig).

### What influences the likelihood that managers use scientific information and other types of information?

Managers reported experience-based evidence to be significantly more easy to access followed by intermediate and scientific evidence sources (F_2, 792_ = 117.8, P < 0.001; Fig 1A). Experience-based evidence was 1.4 and 1.3 times more accessible than scientific and intermediate evidence, and intermediate evidence was considered 1.1 times more accessible than scientific evidence (experience: $\bar{x}$ = 2.29; intermediate evidence: $\bar{x}$ = 1.83; scientific evidence: $\bar{x}$ = 1.63). Managers also reported experience-based and intermediate evidence categories to be more important to decision making, both being 1.1 times more likely to be rated as very important than scientific evidence (F_2, 747_ = 16.11, P < 0.001; Fig 1C; experience and intermediate evidence: $\bar{x}$ = 2.4; scientific evidence: $\bar{x}$ = 2.25). However, the vast majority of managers considered scientific evidence to be “important” or “very important” (S3B Fig).

We found that the characteristics of managers, such as their level of experience, education level, and English fluency played only a weak role in explaining variation in EBDM scores (Experience time: F_1, 222_ = 0.039, P = 0.843; Education level: F_5, 222_ = 0.134, P = 0.984; English fluency: F_3, 222_ = 0.440, P = 0.725; statistics based on the full model; S3 Table). However, managers who had received previous technical training did display significantly higher EBDM scores (β = 0.187, t = 2.239; P = 0.026; statistics based on the most parsimonious model), suggesting they were more likely to be using scientific evidence in their decision making.

We also found that the characteristics of the protected area, such as its size, and type were not influential in the use of scientific evidence by managers (Size: F_1, 222_ = 0.137, P = 0.712; Type: F_1, 222_ = 1.213, P = 0.272). However, the regional location of protected areas may play a small role in the EBDM scores because the difference in AIC was very small when compared to the most parsimonious model (ΔAIC = 0.74), even though the variable was not found to be significant (Location: F_4, 222_ = 1.648, P = 0.163; statistics based on the full model).

We did find a significant effect of greater accessibility of information on EDBM scores (β = 0.353, t = 4.052, P < 0.001), suggesting managers will use more rigorous evidence more frequently when it is available. Likewise, EBDM scores were significantly higher for managers who considered scientific sources to be more important to support their decisions (β = 0.343, t = 2.883; P = 0.004).

Thus, the best model to explain frequency with which managers use rigorous scientific evidence was a combination of the accessibility of evidence, managers’ perceptions of how important scientific evidence is and their technical capacity to interpret that evidence (R^2^ = 0.12; F _3, 237_ = 10.78; P < 0.001; S3 Table). Overall, the explanatory power of all tested models was low, with no model explaining more that 16% of the variation (see S3 Table) suggesting that EBDM scores are influenced by additional variables. The details of the model selection are provided in S3 Table.

Managers reported a range of additional factors to be important to their general decisions about where to source relevant information (S2 Table). Most managers listed the credibility of the source (~80% of the managers), the suitability to the problem (~75%), ease of access (~63%), on-line availability (~60%), and open access (~54%) to be generally important factors when they select relevant information (S2 Table). This confirms that how valuable (important) evidence is and a manager’s ability to access evidence are critical to their ability to achieve evidence based decision making.

## Discussion

Identifying effective management actions based on sound evidence can reduce the uncertainty about whether actions will generate improved conservation outcomes [25]. When considering the regular use of evidence (weekly), we found that Brazilian protected areas managers use scientific evidence less frequently than experience and intermediate sources (Fig 1A). Only a small amount of Brazilian managers access scientific sources on a regular basis (around 10% on a weekly basis; S2A Fig), with these results mirroring studies from English-speaking countries (10% Australia [8], 8% United Kingdom [6]). Like their counterparts in developed countries (e.g. [8,11,26]), Brazilian managers rely more on both their own experience, and to a lesser degree intermediate sources, to guide their day to day actions. Nevertheless, we found that scientific evidence is being used on average less than one a month. We did not ask managers what types of decisions they use the different evidence for and so it is not possible to determine whether this result reflects that managers have greater difficulty accessing scientific evidence, or the frequency with which decisions that require scientific evidence are made. Further study is required to tease out how these two factors interact to influence the use of scientific evidence in management.

When we considered the frequency of use alongside the rigour of the information source (EBDM score), we found that despite the infrequent use of scientific evidence by managers in Brazil, the overwhelming majority do consider science important to support their decisions. This aligns with the results of other studies suggesting that the poor use of science by managers is not a product of their failing to value science [7], suggesting evidence-based management in Brazil appears to be limited by other factors. We also found that managers value the full spectrum of evidence, with only small differences in how managers value the different categories of evidence (Figs 1C and S3B). These findings suggest that similar to protected area managers in other parts of the world [7], Brazilian managers value multiple lines of evidence to support their management decisions.

We also found support for several barriers to the use of scientific information by managers that have been highlighted as important in English-speaking countries, including the accessibility of evidence (Fig 1B) [7] and lack the technical training to interpreting research [6]. This suggests that the concerns that publications are targeted at academics rather than managers [9,27] may be universal to managers regardless of whether they are in developed or developing countries. Other studies have reported that managers are concerned about interpreting research for their management context [11], preferring to discuss findings with scientists [10] or colleagues [6]. This may be contributing to our findings that protected area managers in Brazil are concerned about the credibility and relevance of evidence when selecting information to support their decisions (S2 Table).

While the accessibility of evidence was a major barrier to the use of scientific evidence for Brazilian managers, we also found that even when accessibility was higher, the use of scientific evidence depended on whether managers had training in how to interpret science. These findings suggest that simply improving managers’ access to scientific journals alone will not increase evidence-based management without parallel efforts to improve the technical capacity of managers. Improved access to evidence for managers could be achieved if governments extended existing infrastructure that supports universities to access research, providing access to search engines (e.g., Web of Science) and subscriptions to management relevant journals. However, improving the scientific training of managers may not be in itself be a panacea, with efforts to increase training currently being hampered by high rates of staff turn-over associated with swings in political strategy for protected areas [22]. Therefore, it is difficult for agencies trying to achieve long-term capacity building, and increases the cost of training due to the high proportion of new personnel.

In addition to increasing the scientific literacy of managers as a means to improving evidence-based decision making, the language barrier faced by managers in non-English speaking countries can also be a challenge for these managers understanding the available evidence. English is the international language of science, and this places non-native English speakers at a major disadvantage when accessing and understanding science [17]. Interestingly, while over 50% of managers reported having poor or elementary English skills, only a third considered this a major barrier to the use of scientific evidence. Meanwhile the challenges associated with open access, and the credibility and relevance of science were considered greater barriers to the use of evidence in Brazil (S2 Table). As we did not ask managers about the availability of scientific evidence in their first language (Portuguese), it is unclear how great a barrier language is to accessing relevant literature. This suggests that more research is needed to understand how important language barriers are to evidence-based decision making in non-English speaking countries. This information is critical to developing strategies for improving the use of evidence because increasing assess to literature published in English may be an ineffective strategy if language places an additional barrier to evidence-based decision making for managers in non-English speaking countries [18], such as Brazil.

## Conclusions

This study provides critical insight into the use of different types of evidence by protected area managers outside English speaking countries. Managers in Brazilian protected areas face many of the same challenges in achieving evidence-based conservation management as managers in Australia [8], the UK [6] and other English speaking countries [11]. Just as in developed countries, management decisions in Brazil are rarely guided by the use of scientific evidence, and day to day decisions are much more likely to be guided by experience-based sources. It is encouraging to see that Brazilian managers value science as highly as managers in other countries [7,10], but their efforts to achieve evidence-based decision making are being hampered by poor access to credible, relevant science. Therefore, managers suggested that their use of scientific evidence could be improved by increasing research targeted at high priority management problems and by strategies to increase access to scientific journals. However, our findings highlight that improved access to the literature alone will not achieve greater evidence-based management unless it is coupled with increased technical training to improve their capacity to interpret the findings of research. English fluency did not appear to present a significant barrier to evidence-based management for Brazilian managers relative to the credibility and relevance of evidence, despite many managers indicating that their English was basic. Given that most of the challenges to evidence-based management appear to be similar across countries, many of the solutions may be likewise similar. Therefore, suggestions for greater engagement between managers and scientists to recognize and explore the major challenges to protected areas management could make a significant difference to evidence-based decisions [13]. While managers can benefit from including multiple lines of evidence in their decision-making (e.g. scientific, intermediate, and experience-based evidence [7]), we suggest that providing regular access to scientific evidence, and training in how to use this information, could reduce the uncertainty in decisions and provide greater confidence that management action will improve conservation outcomes.

## Supporting Information

S1 Dataset Frequency of use of sources of information reported by Brazilian managers of protected areas. The dataset does not contain any data that could be used to identify any human participants in this study and frequency of use of sources is coded as in Table 2.
(CSV)

S2 Dataset Accessibility of sources of information reported by Brazilian managers of protected areas. The dataset does not contain any data that could be used to identify any human participants in this study and accessibility of sources is coded as in Table 2.
(CSV)

S3 Dataset Importance of sources of information reported by Brazilian managers of protected areas. The dataset does not contain any data that could be used to identify any human participants in this study and importance of sources is coded as in Table 2.
(CSV)

S4 Dataset Dataset used for modelling evidence based decision making scores as a function of several potential predictors. The dataset does not contain any data that could be used to identify any human participants in this study.
(CSV)

S1 Fig Distribution of Brazilian protected areas. (PDF)

S2 Fig Frequency of use of information sources and evidence categories by Brazilian managers of protected areas. (PDF)

S3 Fig Accessibility and importance of evidence categories reported by Brazilian managers of protected areas. (PDF)

S1 Table Number and size of Brazilian protected areas and questionnaire response rates. (PDF)

S2 Table Factors that may influence managers of Brazilian protected areas in the search for relevant information for management related actions. (PDF)

S3 Table Details of model selection for evidence based decision making scores as a function of several potential predictors. (PDF)

S1 Text Questionnaire submitted to managers of Brazilian protected areas. (PDF)
