# Supplementary material for: Scientific Evidence and Potential Barriers in the Management of Brazilian Protected Areas
Source: PLoS One. 2017 Jan 9;12(1):e0169917. doi: 10.1371/journal.pone.0169917 (PMC5221784; doi:10.1371/journal.pone.0169917)
Supplement: S2 Fig — (PDF) [file pone.0169917.s006.pdf]

Scientific evidence and potential barriers in the management of Brazilian protected areas

Eduardo L. H. Giehl, Marcela Moretti, Jessica C. Walsh, Marco Batalha, Carly N. Cook

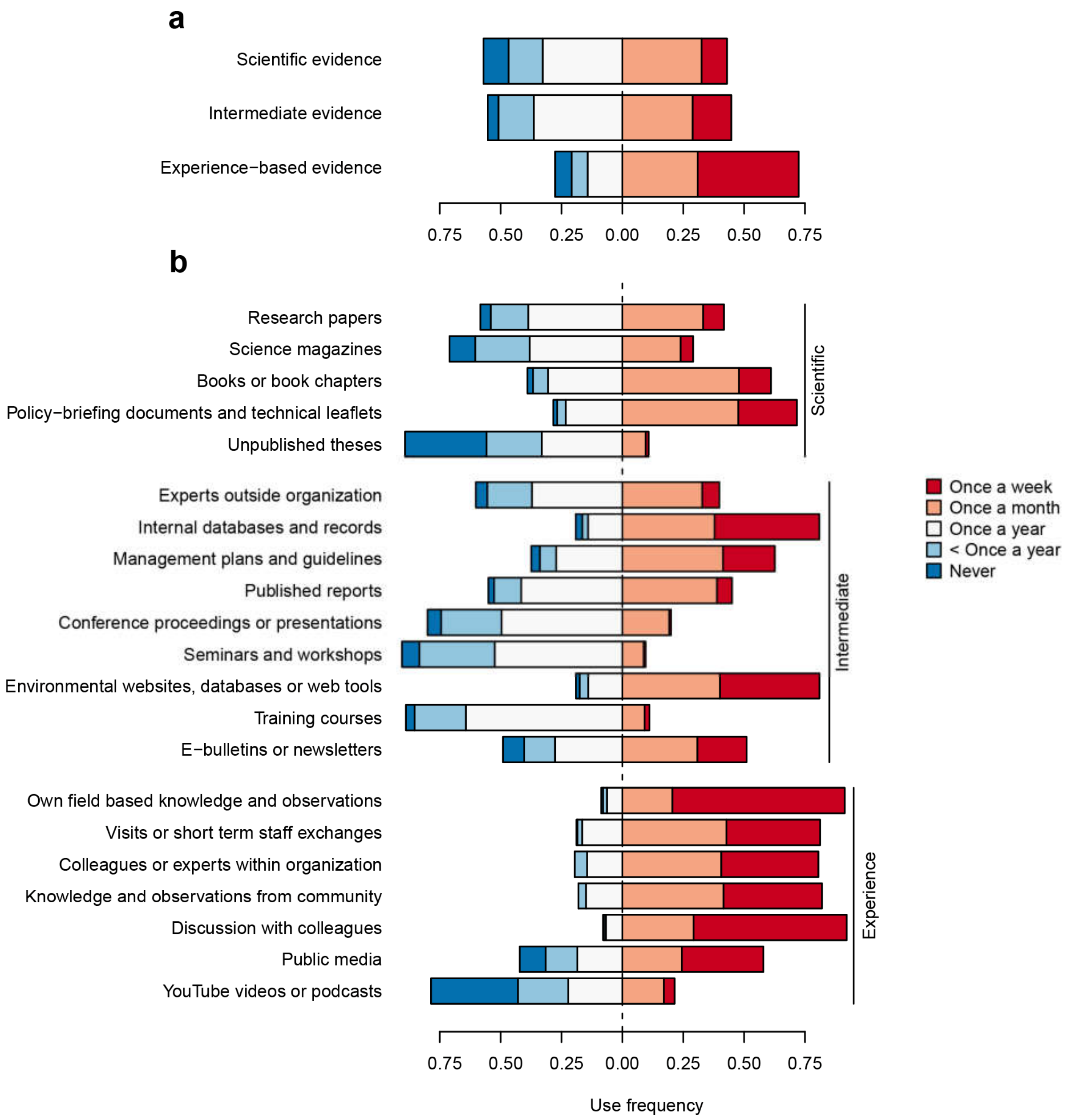

S2 Fig. Frequency of use of information sources and evidence categories by Brazilian managers of protected areas. (a) The combined frequency of use by evidence category (scientific evidence, intermediate and experience). (b) Frequency of use of individual information sources. Bars indicate the proportion of times managers indicated a particular frequency of use category for each information source or evidence category. All horizontal bars add up to 100% and were centred on the division between sources used less than once a month (to the left) and at least once a month or once a week (to the right) to indicate sources or categories used more frequently. For example, less than 10% of all managers use research papers at least once a week, which is much less than their own field-based knowledge and observations (70% of managers; in b). Overall, scientific evidence was less likely to be used on weekly basis (a).
