# Supplementary material for: Scientific Evidence and Potential Barriers in the Management of Brazilian Protected Areas
Source: PLoS One. 2017 Jan 9;12(1):e0169917. doi: 10.1371/journal.pone.0169917 (PMC5221784; doi:10.1371/journal.pone.0169917)
Supplement: S3 Fig — (PDF) [file pone.0169917.s007.pdf]

Scientific evidence and potential barriers in the management of Brazilian protected areas

Eduardo L. H. Giehl, Marcela Moretti, Jessica C. Walsh, Marco Batalha, Carly N. Cook

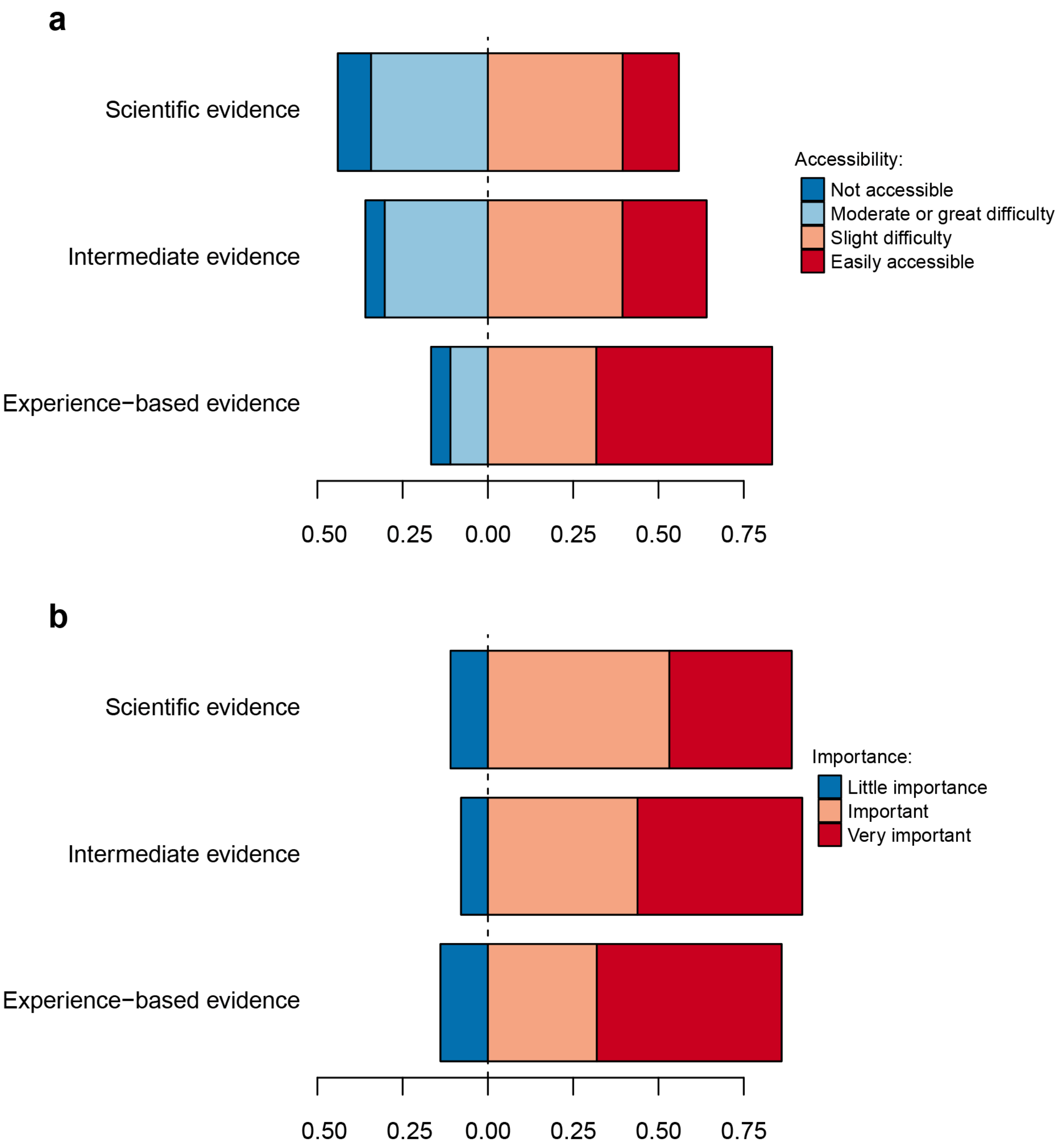

S3 Fig. Accessibility and importance of evidence categories reported by Brazilian managers of protected areas. (a) Accessibility by evidence category (scientific evidence, intermediate and experience). (b) Importance by evidence category. Bars indicate the proportion of times managers indicated a particular accessibility or importance level for each evidence category. All horizontal bars add up to 100% and were centred on the division between categories more easily accessible or at least important. Managers find experience-based evidence easier to access than other categories of evidence (a). Less than 17% of managers reported evidence to be of little importance (b).
