# Supplementary material for: Scientific Evidence and Potential Barriers in the Management of Brazilian Protected Areas
Source: PLoS One. 2017 Jan 9;12(1):e0169917. doi: 10.1371/journal.pone.0169917 (PMC5221784; doi:10.1371/journal.pone.0169917)
Supplement: S1 Table — (PDF) [file pone.0169917.s008.pdf]

Scientific evidence and potential barriers in the management of Brazilian protected areas

Eduardo L. H. Giehl, Marcela Moretti, Jessica C. Walsh, Marco Batalha, Carly N. Cook

S1 Table. Number and size of Brazilian protected areas belonging to distinct protection categories and response rate to our questionnaire. Data on number and area of protected areas is from the National Catalog System of Protected Areas – CNUC (http://www.mma.gov.br/areas-protegidas/cadastro-nacional-de-ucs/; accessed in February, 2015). Response rate shows the number of managers that answered our questionnaires in the indicated type or category (% indicates the number of managers in that type or category from the total number of protected areas in that type or category).

**Type/Category**	**Total**		
**Strict protection**	**Number**	**Area (km^2)**	**Response rate**
Ecological station	91	122,213	25 (27%)
Natural monument	42	1,407	7 (17%)
Park	361	348,088	96 (27%)
Wildlife refuge	32	3,768	2 (6%)
Biological reserve	60	52,531	20 (33%)
Full protection sub-total	*586*	*528,007*	*150 (26%)*
**Sustainable use**			
Forest	104	299,966	32 (31%)
Extractive reserve[1]	90	144,570	35 (39%)
Sustainable development reserve	36	111,293	6 (17%)
Environmental protection area	294	460,922	32 (11%)
Areas of special ecological interest	48	921	4 (8%)
Private reserve of natural heritage	782	5,517	6 (1%)
Sustainable use sub-total	*1354*	*1,023,189*	*83 (6%)*
**Total**	**1940**	**1,551,196**	233 (12%)
