# Supplementary material for: Scientific Evidence and Potential Barriers in the Management of Brazilian Protected Areas
Source: PLoS One. 2017 Jan 9;12(1):e0169917. doi: 10.1371/journal.pone.0169917 (PMC5221784; doi:10.1371/journal.pone.0169917)
Supplement: S2 Table — (PDF) [file pone.0169917.s009.pdf]

Scientific evidence and potential barriers in the management of Brazilian protected areas

Eduardo L. H. Giehl, Marcela Moretti, Jessica C. Walsh, Marco Batalha, Carly N. Cook

S2 Table. Summary of factors that may influence managers of Brazilian protected areas in the search for relevant information for management related actions. Each factor was a yes or no question and factors are shown in order of importance (the number of managers answering "yes" to the question of whether the factor was important). Whether the source is reliable (source credibility) or the information is suitable to the problem at hand were the most important aspects. Conversely, use of jargon or the need for translation had little impact on choosing different sources or information.

Factor	Yes (%)	No (%)
Source credibility	214 (80.1%)	53 (19.9%)
Suitability to the problem	199 (74.5%)	68 (25.5%)
Easy access	167 (62.5%)	100 (37.5%)
On-line availability	160 (59.9%)	107 (40.1%)
Open access	143 (53.6%)	124 (46.4%)
Recommended by colleagues	103 (38.6%)	164 (61.4%)
Available in Portuguese	94 (35.2%)	173 (64.8%)
Easy interpretation	68 (25.5%)	199 (74.5%)
Prior utilization	39 (14.6%)	228 (85.4%)
Easy translation	26 (9.7%)	241 (90.3%)
Absence of scientific jargon	11 (4.1%)	256 (95.9%)
