# Supplementary material for: Scientific Evidence and Potential Barriers in the Management of Brazilian Protected Areas
Source: PLoS One. 2017 Jan 9;12(1):e0169917. doi: 10.1371/journal.pone.0169917 (PMC5221784; doi:10.1371/journal.pone.0169917)
Supplement: S3 Table — (PDF) [file pone.0169917.s010.pdf]

Scientific evidence and potential barriers in the management of Brazilian protected areas

Eduardo L. H. Giehl, Marcela Moretti, Jessica C. Walsh, Marco Batalha, Carly N. Cook

S3 Table. Model selection for evidence based decision making (EBDM) scores as a function of several potential predictors. The results of model selection are based AIC of a multiple regression model and ranked based on increased AIC.

**Model**	**K**	**AIC**	**ΔAIC**	**R^2**
Acc.Sci.Ev + Imp.Sci.Ev + Tech.Cap.[a]	5	434.0	0.00	0.123
Acc.Sci.Ev + Imp.Sci.Ev + Tech.Cap. + PA.Region	9	434.7	0.74	0.151
Acc.Sci.Ev + Imp.Sci.Ev + PA.Type + Tech.Cap. + PA.Region	10	436.5	2.46	0.153
Acc.Sci.Ev + Imp.Sci.Ev + PA.Size + PA.Type + Tech.Cap. + PA.Region	11	438.4	4.43	0.153
Acc.Sci.Ev + Imp.Sci.Ev	4	438.6	4.57	0.099
Acc.Sci.Ev + Imp.Sci.Ev + Exp.Time + PA.Size + PA.Type + Tech.Cap. + PA.Region	12	440.6	6.62	0.153
Acc.Sci.Ev	3	445.1	11.15	0.066
Acc.Sci.Ev + Imp.Sci.Ev + Exp.Time + PA.Size + PA.Type + Tech.Cap. + Eng.Fluency + PA.Region	15	447.1	13.14	0.154
Imp.Sci.Ev	3	451.8	17.77	0.040
Tech.Cap.	3	455.1	21.10	0.026
Acc.Sci.Ev + Imp.Sci.Ev + Exp.Time + PA.Size + PA.Type + Edu.Level + Tech.Cap. + Eng.Fluency + PA.Region[b]	20	457.7	23.69	0.158
~ 1 (Intercept only)	2	459.4	25.44	0.000
PA.Region	6	459.9	25.92	0.032
PA.Type	3	460.1	26.11	0.006
Exp.Time	3	461.4	27.43	0.000
PA.Size	3	461.5	27.46	0.000
Eng.Fluency	5	463.2	29.20	0.010
Edu.Level	7	467.8	33.80	0.009

K: number of model parameters; AIC: Akaike Information Criterion; ΔAIC: difference from the model with the lowest AIC value; R^2: coefficient of determination. Acc.Sci.Ev: Accessibility of scientific sources as indicated by managers; Imp.Sci.Ev: Importance of scientific sources as indicated by managers; Exp.Time: Managers' experience in years; PA.Size: Size of protected area size (ha); PA.Type: Type of protected area; Edu.Level: Managers' education level; Tech.Cap.: Whether managers' had preliminary technical training; Eng.Fluency: Managers' English fluency; PA.Region: Protected area geographic location; ~1: intercept only model.

[a] Most parsimonious model; [b] Full model.
