# Supplementary material for: Scientific Evidence and Potential Barriers in the Management of Brazilian Protected Areas
Source: PLoS One. 2017 Jan 9;12(1):e0169917. doi: 10.1371/journal.pone.0169917 (PMC5221784; doi:10.1371/journal.pone.0169917)
Supplement: S1 Text — (PDF) [file pone.0169917.s011.pdf]

Scientific evidence and potential barriers in the management of Brazilian protected areas

Eduardo L. H. Giehl, Marcela Moretti, Jessica C. Walsh, Marco Batalha, Carly N. Cook

S1 Text. Questionnaire submitted to managers of Brazilian protected areas.

Q0 Dear manager of protected area, we are collecting information for a study that aims to identify the main sources of information used by managers during management of Brazilian protected areas. We believe that effective conservation can be influenced by using distinct information sources during the decision-making process. Besides, communication between managers and researchers has to be optimized and hence we would like to know how you access information. Based on your answers we would like to build a set of recommendations for the government and the scientific community. This study is addressed to all managers registered as protected areas within the Brazilian Environment Ministry.

We therefore kindly ask you to voluntarily answer a short questionnaire that will require around 15 min to fulfill. Your participation will help us in data collection and to finish our study. All answers will be collected anonymously and confidentially, and will not be directly published. At the end of the questionnaire you can fill in your e-mail in case you want to be informed about the results of the study. We would also ask you to forward this questionnaire to your colleagues, enabling us to increase our sample size and to better describe the current situation in Brazilian protected areas. Our team includes researchers from the São Carlos Federal University, Cambridge University, and Monash University. If you have questions or comments about our research, please contact us by e-mail (ufscarpesquisa@gmail.com). Sincerely,

Q1 I have read and understood the information above and in the Project Information Sheet and am willing to participate in this study.

- ❍ Yes
- ❍ No

Q2 You have chosen to not participate in this study. Thank you for your time. If you do wish to continue the survey, press 'Back', which will take you to the previous page.

Q3 Instructions: We are going to ask you 17 questions and expect that fulfilling the survey will take around 15 min. You may use ‘Back’ and ‘Next’ buttons to navigate between pages. Please do not use your browser buttons because they can generate errors. To submit the questionnaire, you will have to fulfill it up to the last page. You may also come back and end filling the questionnaire in the next days unless you clean your browser cache.

Please mind that this questionnaire will expire in 30 days when no more answers are going to be collected. Click 'Next' to start answering the questionnaire.

Q4 What category of protected area do you work in?

- ❍ Area of environmental protection
- ❍ Area of relevant ecologic interest
- ❍ Ecological station
- ❍ National or state forest
- ❍ Natural monument
- ❍ National or state park
- ❍ Biological reserve
- ❍ Sustainable development reserve
- ❍ Extractive reserve
- ❍ Natural heritage private reserve
- ❍ Other ____________________

Q5 In which Brazilian state is the protected area located?

Q6 What is the size of the protected area (in hectares) you work in?

Q7 Which of the below options best describes your role in the protected area? (You may select more than one option)

- ❑ Implementing conservation and management actions
- ❑ Taking decisions about which actions are more satisfactory
- ❑ Advising conservation professionals and managers about management issues
- ❑ Other ____________________

Q8 Have you been previously trained before taking the position? If you had a preliminar training, please enter a short description about the type of training and in which institution you were trained.

- ❍ Yes/details ___________________
- ❍ No

Q9 For how many years have you been working in protected areas?

Q10 Please specify the highest education level you have achieved so far:

- ❍ No formal education
- ❍ Primary school education
- ❍ Secondary school education
- ❍ Technical school education
- ❍ Diploma
- ❍ Masters degree
- ❍ Doctorate
- ❍ Other ___________________

Q11 How well can you speak, read and understand English?

- ❍ Not at all
- ❍ Not well
- ❍ Well
- ❍ Very well

Q12 Imagine that you have a question about an environmental or conservation management issue. Where are the first five places you go to search for information about this question? (please select at most five options)

- ❑ Web-based general search engines e.g. Google
- ❑ Open access web-based database of scientific publications, e.g. Google Scholar
- ❑ Subscription web-based database of scientific publications, e.g. Web of Science, Scopus
- ❑ Internal databases or intranets

- ❑ Specific websites, online environmental databases or tools
- ❑ Electronic library catalogue or browsing in the library
- ❑ Personal collection of books, reports and electronic material
- ❑ Ask experts or advisors
- ❑ Recommendations of literature from colleagues
- ❑ Social or professional web-based networks, e.g. Twitter, Facebook, Linked-In
- ❑ Conferences or professional meetings
- ❑ E-bulletins, newsletters or magazines (electronic or paper)
- ❑ Training programs, courses or seminars
- ❑ Other ___________________

Q13 Which of the following factors are important to you when choosing information about conservation management? (please select a maximum of five)

- ❑ The type of information needed
- ❑ Easy and fast access
- ❑ Free to access
- ❑ Easy to interpret
- ❑ Lack of jargon
- ❑ Available in Portuguese
- ❑ Easy to translate
- ❑ Available online
- ❑ Recommended by colleagues
- ❑ Previous experience of using it
- ❑ Source credibility
- ❑ Other ___________________

Q14 Which sources of information do you have access to? Please select the most appropriate option for each type of information source.

We want to know how easy is it for you to obtain each resource, even if you don't use it often. **Not accessible** means you are unable to obtain the information directly or indirectly. **Accessible with moderate or great difficulty** means it requires a lot of effort, time or money to access the information, such as having to ask multiple people for permission to access the information or having to purchase it. **Accessible with slight difficulty** means you can access the information indirectly, but are required to take an extra step to obtain it, for example you have to ask someone else to send you the information or you have to go to a library to locate he information. Easily accessible means you can obtain the information immediately, for example in a personal library collection or from an online resource.

Source	Not accessible	Accessible with moderate or great difficulty	Accessible with slight difficulty	Easily accessible	Don’t know
Scientific research papers	❍	❍	❍	❍	❍
Books or book chapters	❍	❍	❍	❍	❍
Published reports	❍	❍	❍	❍	❍
Scientific journals	❍	❍	❍	❍	❍
Conference proceedings or presentations	❍	❍	❍	❍	❍
Management plans, working manuals and guidelines	❍	❍	❍	❍	❍
Specific environmental websites, databases or web tools	❍	❍	❍	❍	❍
Internal databases, archives and records	❍	❍	❍	❍	❍
Policy-briefing documents and technical information leaflets	❍	❍	❍	❍	❍
E-bulletins or newsletters	❍	❍	❍	❍	❍
Training courses	❍	❍	❍	❍	❍

Seminars and workshops	❍	❍	❍	❍	❍
YouTube videos or podcasts	❍	❍	❍	❍	❍
Advice from experts or scientists outside organization	❍	❍	❍	❍	❍
Advice from colleagues or experts within organization	❍	❍	❍	❍	❍
Public media, e.g. newspapers, television, films, radio	❍	❍	❍	❍	❍
Own field based knowledge, observations and experience	❍	❍	❍	❍	❍
Local knowledge and observations from community members	❍	❍	❍	❍	❍
Site visits or short term staff exchanges	❍	❍	❍	❍	❍
Informal discussion with colleagues	❍	❍	❍	❍	❍
Unpublished theses	❍	❍	❍	❍	❍
Other	❍	❍	❍	❍	❍

Q15 During management actions within the protected area, please indicate how frequently do you use each of the following resources. Besides, point out how important do you think that each source is during your decisions.

	Frequency of use					Importance		
**Source**	**At least once a week**	**At least once a month**	**At least once a year**	**Less than once a year**	**Never**	**Little importance**	**Important**	**Very important**
Scientific research papers	❍	❍	❍	❍	❍	❍	❍	❍
Books or book chapters	❍	❍	❍	❍	❍	❍	❍	❍
Published reports	❍	❍	❍	❍	❍	❍	❍	❍
Scientific journals	❍	❍	❍	❍	❍	❍	❍	❍
Conference proceedings or presentations	❍	❍	❍	❍	❍	❍	❍	❍
Management plans, working manuals and guidelines	❍	❍	❍	❍	❍	❍	❍	❍
Specific environmental websites, databases or web tools	❍	❍	❍	❍	❍	❍	❍	❍

Internal databases, archives and records	❍	❍	❍	❍	❍	❍	❍	❍
Policy-briefing documents and technical information leaflets	❍	❍	❍	❍	❍	❍	❍	❍
E-bulletins or newsletters	❍	❍	❍	❍	❍	❍	❍	❍
Training courses	❍	❍	❍	❍	❍	❍	❍	❍
Seminars and workshops	❍	❍	❍	❍	❍	❍	❍	❍
YouTube videos or podcasts	❍	❍	❍	❍	❍	❍	❍	❍
Advice from experts or scientists outside organization	❍	❍	❍	❍	❍	❍	❍	❍
Advice from colleagues or experts within organization	❍	❍	❍	❍	❍	❍	❍	❍

Public media, e.g. newspapers, television, films, radio	❍	❍	❍	❍	❍	❍	❍	❍
Own field based knowledge, observations and experience	❍	❍	❍	❍	❍	❍	❍	❍
Local knowledge and observations from community members	❍	❍	❍	❍	❍	❍	❍	❍
Site visits or short term staff exchanges	❍	❍	❍	❍	❍	❍	❍	❍
Informal discussion with colleagues	❍	❍	❍	❍	❍	❍	❍	❍
Unpublished theses	❍	❍	❍	❍	❍	❍	❍	❍
Other	❍	❍	❍	❍	❍	❍	❍	❍

Q16 How much time do you regularly spend Reading scientific articles per week?

- ❍ Less than 1 hour
- ❍ 2-4 hours
- ❍ 5-7 hours
- ❍ 8-10 hours
- ❍ 11-13 hours
- ❍ 14-16 hours
- ❍ More than 17 hours

Q17 Do you use scientific information either during planning or taking actions within the protected area.

	Always	Most of the time	Almost never	Never	Don't know	If possible, provide an example
Results of scientific publications are tested or used within the protected area	❍	❍	❍	❍	❍	
Planning of management actions is based on publications in administration, management and planning fields	❍	❍	❍	❍	❍	

Q18 List factors that you believe are more limiting while trying to use the results of scientific research.

Q19 Please answer the following questions, selecting the most appropriate answer for each statement.

	Agree	Disagree	Don't know	Why?
Results of scientific publications are difficult to interpret.	❍	❍	❍	
I believe that I could benefit from better access to scientific publications.	❍	❍	❍	
Scientific publications have a big importance on my decisions.	❍	❍	❍	

Q20 Regarding scientific articles, answer the following questions:

	Yes	No	Give examples or explain why
Do you believe there is room to bridge theoretical scientific knowledge and practical actions?	❍	❍	
Are there any researchers with ongoing studies in the protected area you work in?	❍	❍	
If there are/were any research studies in the protected area you work in, do you generally have access to the results of the studies?	❍	❍	

Q21 Please answer the following questions regarding threatened or invasive species within the protected area you work in. Please provide additional details wherever possible.

	Yes	No	Additional details
Are there any threatened species hosted in the protected are you work in (how many and examples)?	❍	❍	
Is there monitoring of populations from any of the threatened species within the protected area you work in (if there is, please indicate the main source for the monitoring: management plan, own experience, or scientific publications)?	❍	❍	
Are there any invasive species in the protected area you work in?	❍	❍	
Are there strategies for eradication or controlling the growth of invasive populations (if there is, please indicate the main source for the monitoring: management plan, own experience, or scientific publications)?	❍	❍	
